# Searching for functional gene modules with interaction component models

**DOI:** 10.1186/1752-0509-4-4

**Published:** 2010-01-25

**Authors:** Juuso A Parkkinen, Samuel Kaski

**Affiliations:** 1 Helsinki Institute for Information Technology HIIT and Adaptive Informatics Research Centre, Department of Information and Computer Science, Helsinki University of Technology, P.O. Box 5400, FI-02015 TKK, Finland; 2 Department of Computer Science, P.O. Box 68, FI-00014, University of Helsinki, Finland

## Abstract

**Background:**

Functional gene modules and protein complexes are being sought from combinations of gene expression and protein-protein interaction data with various clustering-type methods. Central features missing from most of these methods are handling of uncertainty in both protein interaction and gene expression measurements, and in particular capability of modeling overlapping clusters. It would make sense to assume that proteins may play different roles in different functional modules, and the roles are evidenced in their interactions.

**Results:**

We formulate a generative probabilistic model for protein-protein interaction links and introduce two ways for including gene expression data into the model. The model finds interaction components, which can be interpreted as overlapping clusters or functional modules. We demonstrate the performance on two data sets of yeast *Saccharomyces cerevisiae*. Our methods outperform a representative set of earlier models in the task of finding biologically relevant modules having enriched functional classes.

**Conclusions:**

Combining protein interaction and gene expression data with a probabilistic generative model improves discovery of modules compared to approaches based on either data source alone. With a fairly simple model we can find biologically relevant modules better than with alternative methods, and in addition the modules may be inherently overlapping in the sense that different interactions may belong to different modules.

## Background

Searching for hypotheses about functional gene modules, co-regulated sets of genes and protein complexes, has been under intensive research effort given the current high-throughput data acquisition methods. Traditionally only a single data type, gene expression or protein-protein interaction (PPI) data is used (see for example [1,2]). Recently also methods for combining relational interaction data and functional gene expression data have been studied, for example [3,4].

Ulitsky and Shamir [5] recently used similarities between gene expression patterns as a kind of interaction data between proteins. They combined these interactions with protein-protein interaction measurements in order to seek *Jointly Active Connected Subnetworks *(JACS). Their novel computational method called *Matisse *found biologically relevant modules better than a set of earlier methods (e.g. Co-clustering [6] and CLICK [7]).

Another recent method [8] uses a protein-protein interaction network to form prior constraints on the clustering of gene expression data. The method is an extension of Markov random fields, called *hidden modular random fields *(HMoF). The constraints improved performance in the task of finding functionally enriched modules, compared to using either data source alone. The HMoF and Matisse have recently been compared [5,8] to a wide set of state-of-the-art methods, and hence they can be considered to be the best current methods.

We formulate a generative probabilistic model for combined gene expression and protein interaction data. The model thus naturally includes a noise model for both data types, which is missing from many other methods, such as Matisse. Protein-protein interaction data is known to be notoriously noisy [9], and even manual curation may not be able to remove all uncertainties in the data. The specific probabilistic model family also allows nodes to inherently belong to several clusters at the same time, so we can interpret the results as overlapping functional modules. This overlap goes beyond standard mixture models to the so-called component models, in this case assuming that each interaction belongs to a specific module, and hence proteins to multiple modules. This is biologically sensible, as many genes and proteins are known to participate in multiple functions, and hence functional modules can overlap with each other. This feature is missing from both HMoF and Matisse.

The methods we propose here are based on a recently introduced generative model for graphs [10]. It assumes that the links, or here molecular *interactions*, can be explained by a set of latent components. In this paper we introduce ways of incorporating functional data related to the nodes, that is, the genes or proteins, into the model. The underlying assumption is that interacting genetic complexes or modules share functional properties in addition to being strongly interconnected. Evidence for this feature has been found in humans [11] and yeast [12]. In the paper we use the notions "interaction" and "link" interchangeably, as well as "gene" and "protein", assuming that there is a one-to-one relation between them.

## Results and Discussion

### Methods and data

Our models are based on the Interaction Component Model (ICM) [10,13]. We introduce two extensions for combining PPI and gene expression data in the model framework. In the first model variant (ICMg1) the expression data is transformed into additional interactions and in the second one (ICMg2) the expression is included in the generative process. The models are applied on a PPI data set from the yeast *Saccharomyces cerevisiae*, combined with two different gene expression sets in order to seek functional gene modules. We compare our methods with the recently introduced HMoF [8] and Matisse [5]. Both methods combine interaction and expression data and have been proven to outperform a set of earlier methods that use only one of the data sources in the task of finding gene modules. The basic ICM that uses only the protein interaction data is also included in the study for comparison. All the models give as a result a clustering for the genes.

Our models provide, for each interaction membership, probabilities over the components. These probabilities can be interpreted as overlapping clusters where each node may be assigned to multiple clusters. We demonstrate this feature with an artificial data case study. However, as the other methods provide only single assignments of nodes to clusters, we transform the component memberships into a crisp clustering for the actual biological comparisons. This is done by simply assigning each gene to the most probable cluster.

Matisse differs from the other methods in the sense that it leaves some genes out from the clustering and also infers the number of clusters automatically. Due to the probabilistic nature of all the models, the number of clusters could be set automatically in several well-justifiable ways, such as cross-validation and different types of information criteria (see e.g. [14] for standard model selection methods). For our methods a natural option would be to use a Dirichlet Process prior for the component distribution. Dirichlet Process is a common non-parametric prior for estimating the number of components based on the data (Teh, Y. W.: Dirichlet Processes, submitted to Encyclopedia of Machine Learning).

However, since implementation of comparable model complexity control methods would be laborious in practice for some of the methods, we fix the number of clusters of the other methods to the median of 20 Matisse runs to bias the results in favor of Matisse, to make sure that the result is not due to the additional degrees of freedom we have in choosing the cluster sizes. We ran each method 20 times to obtain confidence intervals, resulting in a different set of clustered genes for each Matisse run.

### Finding biologically relevant modules

Our goal is to find biologically meaningful functional gene modules by clustering genes based on PPI and gene expression data. Because only a small fraction of the true gene functions is known, the validation of the obtained clustering is not straightforward. The Gene Ontology (GO) [15] annotation database is commonly used as a reference set for model validation, and we will use it as well.

In the first part of the model evaluation we choose *a priori *a set of standard gene classes from the GO. We then use the classes for so-called external validation, by measuring how well the obtained clustering corresponds to the known classes. The motivation is that although the external classification is an imperfect description of the data, a better clustering should reflect it somewhat more. As a goodness measure we use perplexity of predicting the gene classes given the obtained clusters. We additionally complement the analysis with the commonly used GO enrichment analysis to find how well our clusters correspond to other known gene annotations. Finally, we validate the modules in terms of how well they overlap with known protein complexes.

### Agreement with standard gene classes

**Figure 1:**
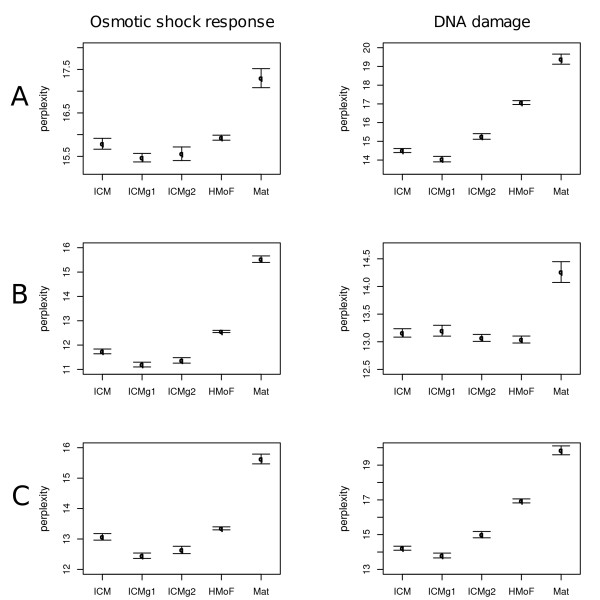
**Agreement with standard gene classes**. Perplexities were computed for all methods for two datasets: Osmotic shock response, left) and DNA damage, right). We used three standard gene cluster sets: **(A) **All genes, **(B) **Common genes, **(C) **Total genes. The 2SE error bars are over 20 runs. Lower perplexity score is better. *Mat *is Matisse.

We computed perplexities comparing the module results to three different sets of standard gene classes, based on 1) all genes in the data, 2) those genes common to all Matisse runs and 3) those genes appearing in at least one Matisse run. The results are shown in Figure 1.

The perplexity results show that our three new methods (ICM, ICMg1 and ICMg2) basically outperform the two comparison methods. Difference to Matisse is clear, which could in principle be due to Matisse leaving nodes out from the clustering. We checked that this is not the case by using only those genes that appear in the Matisse runs, which should give Matisse an advantage in this sense, but the difference still remains clear (Figures 1B and 1C). Difference to HMoF is smaller, and in one case HMoF performs equally well to our methods.

Out of the ICM models, the variant where expression data is included as further links (ICMg1), is the best in all cases except one. Somewhat surprisingly, ICM that uses only PPI data seems to be better than ICMg2 on the DNA damage dataset.

### Gene Ontology enrichment analysis

**Figure 2:**
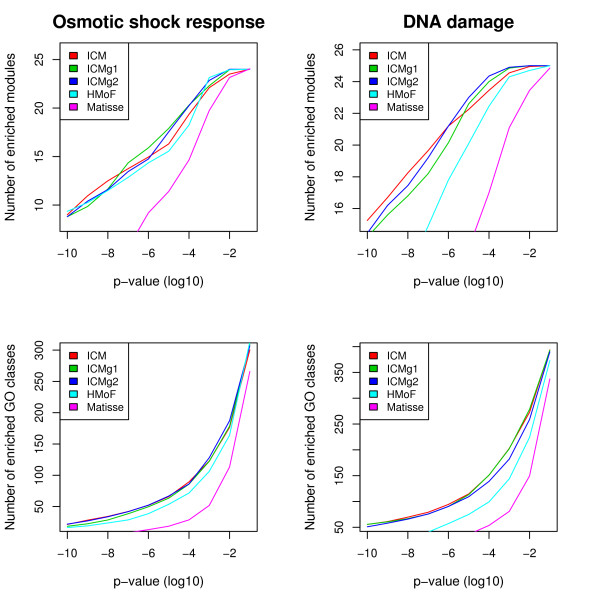
**GO enrichment results**. The number of enriched modules and GO classes as a function of the hypergeometric p-value cutoff. Top row: The number of modules in which at least one GO class is enriched. Bottom row: The number of GO classes enriched in at least one module. Left: Osmotic shock response data. Right: DNA damage data. All values are means over the 20 runs. More enrichments is better.

We complemented the validation with a commonly used Gene Ontology enrichment analysis. Figure 2 shows the number of enriched modules and GO classes as a function of the cutoff p-value for enrichments. Matisse does not perform as well as the other methods in the enrichment analysis. The other four methods perform about equally well in the Osmotic shock response data set, but in the DNA damage data set our methods outperform HMoF as well.

### Protein complexes

**Figure 3:**
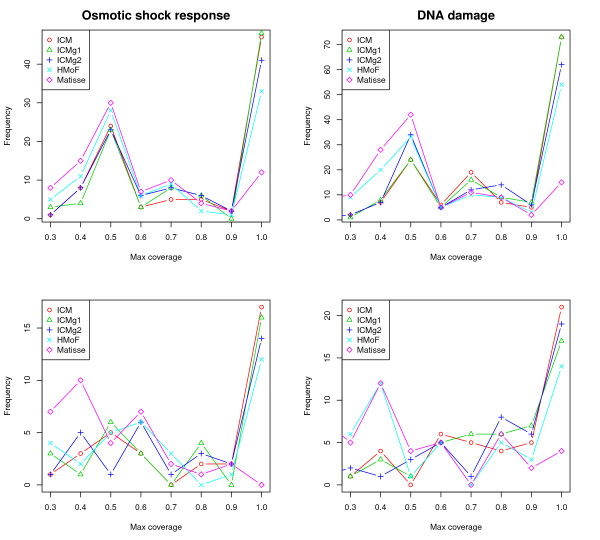
**Protein complex coverage**. The number of protein complexes (y-axis) with a specific degree of coverage (x-axis). Top: complexes with at least 2 proteins, bottom: complexes with at least 5 proteins, left: Osmotic shock response data, right: DNA damage data. The right end of the curves is the important location.

We finally measured how well the found modules match with known protein complexes. From the results, shown in Figure 3, it is evident that the first four methods find a significant amount of the protein complexes with the ICM variant outperforming HMoF to some extent, whereas Matisse's performance is clearly worse. We checked that this was not due to Matisse leaving part of the genes out of the clustering: the difference to the other methods was still clear when genes left out by Matisse were discarded from the other methods too, before or after their analysis (results not shown).

### Demonstration of a sample module

**Figure 4:**
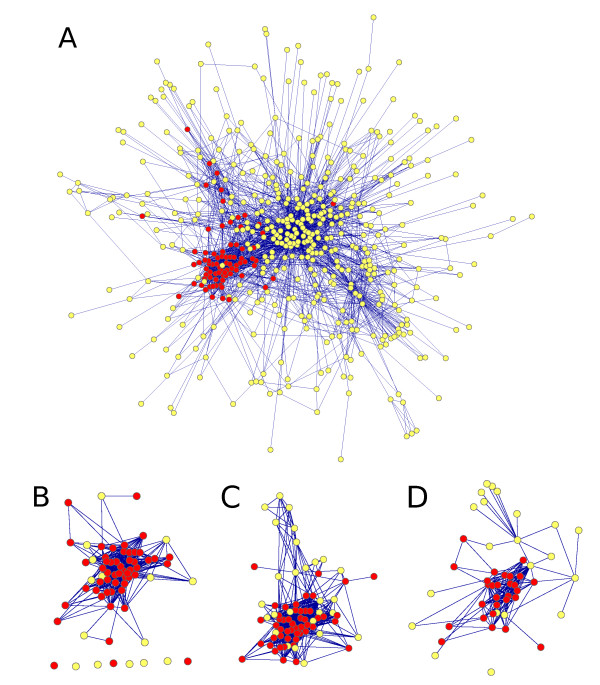
**Demonstration of a sample module found by the methods**. **(A) **Shows a connected subgraph of the PPI network with 520 genes and 2633 protein interactions between them. Out of these, 99 genes belong to the Gene Ontology class *Ribosome biogenesis *(colored red in the graph) and form a clearly visible subgraph. Graphs **(B-D) **are subgraphs of (A), corresponding to a module found by methods ICMg2, HMoF and Matisse, respectively. Again, red color indicates that the gene belongs to the *Ribosome biogenesis *GO class.

Figure 4 shows a subgraph of the PPI network and three modules found by the different methods, visualized with Cytoscape http://www.cytoscape.org/. From the subgraphs (B-D) it is evident that all the modules contain a significant number of genes belonging to the GO class *Ribosome biogenesis*. However, the ICMg2 module includes only a couple of genes that do not belong to the GO class, whereas for the other methods this number of "false positives" is clearly larger. In addition, three from the eight genes included in the ICMg2 module that are not connected to the other genes belong to the same GO class, illustrating the ability of ICMg2 to successfully utilize the gene expression data in addition to the PPI network.

### Overlapping modules

We have now shown empirically that the ICM methods perform well in the task of finding biologically relevant functional modules, outperforming two recently introduced methods that were designed for this particular task. We now further study the ability of our the methods to model and detect overlapping modules, that is, multiple assignments of nodes to modules.

We carry out the study on artificial data to guarantee that the ground truth is available. We generated a network of 10 modules with 10 nodes each. Links were generated with inter- and intra-module link probabilities 0.01 and 0.9, respectively. Additional links were then generated between the modules such that all modules shared two nodes with at least one other module. The resulting network contained 10 partially overlapping modules with 10-12 nodes each, and in total 599 links. For this data we can compare the ICM and HMoF with full weighting on network data (*ω *= 1). Matisse can not be run with network data only. We set the number of modules for both methods to 10.

We evaluated the predicted node assignments obtained by the methods against the known assignments, in which most nodes are assigned to exactly one module with probability 1, and those shared by two modules belong to each with probability 0.5. For ICM we use the probabilistic memberships *p*(*z*|*i*) (equation 8 in Methods) and for HMoF we simply have the binary assignments.

The distance measure in the comparisons was the simple Euclidean distance between the node membership distributions. Both methods were run 10 times, but the variation was vanishingly small. The distances were 0.052 for ICM and 0.127 for HMoF, suggesting that ICM benefits from the probabilistic assignments of nodes to modules. We note that ICM with the memberships binarized gives exactly the same results as HMoF.

## Conclusions

We have used a generative model of relational data to take into account the uncertainty in PPI data when searching for overlapping functional modules of genes. We have also introduced two approaches for combining gene expression data with the interaction data. Experiments with data from the budding yeast suggest that generative modeling of combined expression and interaction data is advantageous. The proposed models outperformed the state-of-the-art methods, HMoF and Matisse, which in turn have recently been shown to outperform the relevant alternatives.

In its current form, ICM is able to detect modules with relatively large number of missing links. What is missing, however, is a suitable noise model for inter-cluster links. This extension will be considered in the future.

We noticed that including the gene expression data in the analysis resulted in most cases only in minor improvements. Others have come to similar conclusions [8,16]. There are many possible explanations for this. The effect might really be biological; the functional gene modules may tend to be interconnected more often than they do share similar expression profiles. The GO annotations used for evaluation may also be biased towards PPI data.

Another reason can also be that the way the models treat gene expression should still be improved. For example, our approaches or the k-means-based modeling used by Shiga et al. [8] have chosen to be relatively simple for computational reasons. Our model variant ICMg2 assumes that there are global components responsible for the behavior of a large group of genes under a large number of different conditions, which is probably an oversimplification. Still it seems to work to some extent. Taking the variation across the different conditions more carefully into account could improve the benefits of using expression data.

It is also possible that only a small part of the expression data is actually relevant for the task. For example, in our model variant ICMg1 we effectively use only the most correlated pairs of gene expression profiles, and the results seem to consistently outperform the plain ICM model, which does not use the expression.

We only recently found out about a related method called DetMod [17] that addresses many important aspects of detecting functional modules, such as automated detection of the number of modules and potential overlap of modules. However, DetMod does not have a noise model for the protein interaction data and hence is dependent on its good quality. In future work the methods should be compared and best insights of both combined.

Finally, we believe that the ability of modeling functional "roles" of proteins, by assuming that their different interactions may belong to different modules, is a promising direction for future research. ICM is able to find such roles, as we demonstrated on artificial data, and is empirically better than alternatives even when this property is not utilized. Next we should study the discovered roles in more detail.

## Methods

### Component models for combining protein interaction and gene expression data

#### Interaction component model

**Figure 5:**
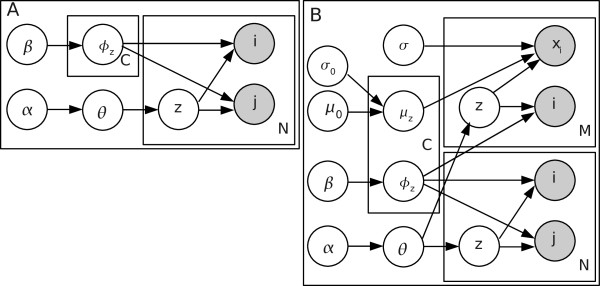
**Plate diagrams of the two Interaction Component Models**. **(A) **Plain ICM. Each interaction is assumed to have been generated from a component *z*, by sampling the end points *i *and *j *independently from the multinomial distribution *ϕ*_*z*_. **(B) **Extension (ICMg2) with gene expression data *x*_*i *_in the nodes; the lower part of the plate is the same as in (A). Node data *x*_*i *_are assumed to have been generated from the same components *z *as the interactions, by sampling each node *i *in the data from *ϕ*_*z *_and then its gene expression profile from the normal distribution *N *(*μ*_*z*_, *σ*^2^*I*). Symbols: multinomial distribution *θ *over components, component-specific multinomial distributions *ϕ*_*z *_over nodes, distribution hyperparameters *α *and *β*, prior and component-specific means *μ*_0 _and *μ*_*z*_, prior and shared noise standard deviations *σ*_0 _and *σ*, number of components *C*, links *N *and nodes *M*.

The generative model we use for the interactions [10,13] assumes that each link or interaction comes from a latent component, each component having a characteristic distribution over nodes. The links are generated (Figure 5A) by first choosing the component *z *based on the multinomial distribution parametrized by *θ*, and then choosing the endpoints *i *and *j *of the link according to the multinomial distribution *ϕ*_*z *_of the component *z*. Note that in the generative process each link belongs to one component; nodes may belong to several.

This model has been proven effective in detecting meaningful communities in large social networks [13]. Here we use the same model structure in searching for functional modules among protein interaction networks, where it is capable of handling uncertainties in the PPI data. Next we introduce two ways of extending the model to take into account functional data available about the nodes, here gene expression data, which is supposed to improve the detection of functional modules.

#### First variant (ICMg1): Transforming expression into links

A very simple way of including functional data about the genes is to transform the data into links that describe the functional similarity of the genes, and to include those links into the graph. We compute the Pearson correlation of expression for each pair of genes, and treat as additional links all pairs where the correlation exceeds 0.85 (the same cutoff value as in [16]). Then we simply pool the original PPI links and the new expression links together. The motivation is that both the existence of protein-protein interactions and potential co-regulation inferred from the correlation links give evidence of functional relatedness of the genes. This approach is similar to the one used in [5], apart from the fact that we do not make any difference between the two types of links. This model variant is denoted as ICMg1, g referring to gene expression.

A question arises whether negative correlation should be taken into account as well, for a strong negative correlation could also be an indication of functional similarity of genes. Here we have omitted such correlations, as they were practically absent (0 and 20 gene pairs with correlation below -0.85 in the osmotic shock response and DNA damage datasets, respectively).

#### Second variant (ICMg2): Generative model for expression data

Another way to incorporate the gene expression data is to assume that the same components generate the gene expression data as well (Figure 5B). This leads to modules which are both strongly interconnected and share similar expression profiles. In practice, the component *z*, from which the gene expression profile *x*_*k *_is generated, is assumed to have been sampled from the same distribution *θ*_*z *_as the component for the links. Note that for computational reasons we have simplified the model by not constraining it with the known fact that each node has exactly one gene expression profile. This model variant is denoted as ICMg2.

#### Parameters

The models have some tunable parameters which affect their performance. All these parameter values were chosen *a priori *and not optimized. Our ICM models have two hyperparameters controlling the component distribution and node distributions within components. Based on earlier studies we set the hyperparameter values to *α *= 10 and *β *= 0.01 (see Figure 4). The model variant ICMg2 has three additional hyperparameters for generating the expression data, which we set to *μ*_0 _= 0,  = 1 and *σ*_2 _= 0.1 to describe small variations around the base value of zero.

The number of clusters for all other methods than Matisse was set to the median of 20 Matisse runs on both datasets, resulting in 24 and 25 clusters in the osmotic shock response and DNA damage data sets, respectively. HMoF has a weight parameter *ω *defining the relative weighting between the expression and network data in the model. This was fixed to *ω *= 0.2 as in the original paper [8]. Matisse was run with the default parameters given in its implementation.

#### Estimation

We estimated our models with collapsed Gibbs sampling [18], where some of the parameters are integrated out and latent variables are sampled. Here the latent variables give the assignments of the links (and node data in ICMg2) to the components. Other estimation methods, such as EM, would be straightforward to implement, but we are worried about overfitting which is dealt with nicely in collapsed Gibbs using suitable priors. Fortunately, the specific collapsed Gibbs is reasonably simple and fast, as explained below. This efficient computation is possible if we choose conjugate Dirichlet priors. Compared to other potential modeling assumptions such as Gaussianity, the combination of multinomials and Dirichlets naturally matches better the discrete data domain.

Joint probability of the basic ICM (Figure 5A) is as follows:(1)

Where *L *is the set of links in the data and *z *are their assignments, *D*_1_(*α*, *β*) is a normalizing constant arising from the Dirichlet priors, *θ *is the global distribution over the components and *ϕ*_*z *_are the component-wise distributions over the nodes *i*, *n*_*z *_is the count of links assigned to component *z*, and *q*_*zi *_counts the component-node-co-occurrences, and *C *and *M *are the number of components and nodes, respectively.

Marginalizing over *θ *and *ϕ*, and separating the effect of one link, holding all other link assignments fixed, results in the following sampling equation for each link:(2)

where *i*_0 _and *j*_0 _correspond to the end points of the left-out link and {*L*}' and {*z*}' are all the other links and their assignments, respectively. In the sampling algorithm we leave one link out at a time and sample a new component *z*_0 _for it with the probabilities (2). This involves bookkeeping of counts *n *and *q *and each link's component assignment.

For ICMg2 (Figure 5B) joint probability includes the link probabilities equivalent to those of ICM, and additionally normal distributions for the gene expression profiles:(3)

where *D*_2 _is again a normalizing constant, *m*_*z *_is a count of the node data points  assigned to each component,  and  are the component-specific and prior node data means, respectively, and *V *and *V*_0 _are the data and prior covariance matrices, respectively.

Now we marginalize over the component-wise expression means  in addition to *θ *and *ϕ*. Separating the effect of one link is analogous to ICM, resulting in the following sampling equation:(4)

Separating the effect of one node and its expression profile in turn results in the following sampling equation:(5)

where posterior covariance matrix *S *is(6)

and posterior mean *A *is(7)

The notation *A' *in (5) denotes that *A *is computed without the effect of the left-out node data. The *S' *is computed analogously.

For ICMg2, one sampling iteration includes sampling the component assignments for each link and for each node's expression profile. This involves bookkeeping of counts *n*, *m *and *q*, component-wise datasums , and component assignments of the links and nodes.

Component memberships of nodes can be estimated in both model variants by the following equation:(8)

In the biological experiments we transform these memberships into a crisp clustering by simply assigning each gene to the most probable cluster (component *z *that maximizes the probability *p*(*z*|*i*)). Additionally, we evaluated the ability of our model to capture multiple cluster assignments from artificial data. For this, we used the probability *p*(*z*|*i*) as such.

In the sampling we first ran 19000 burn in iterations, after which we took 20 samples with an interval of 50 iterations. Clustering results were then obtained by averaging over these samples.

### Datasets

#### PPI and gene expression data

Our PPI data set is obtained by pooling the yeast data sets of [5] and [16], which are originally obtained from various public databases. Our first gene expression data set is the osmotic shock response (OSR) set of [19] and the other one is a DNA damage (DNAD) set of [20]. Since the implementations of all methods do not support missing samples in the sense that either expression or PPI links would be completely missing from some genes, we analyzed subsets without such missing data.

We obtain two combined data sets, one with 1711 genes, 10250 interactions and 133 observations of gene expression (OSR), and another with 1823 genes, 12382 interactions and 52 gene expression observations (DNAD). Pooling the expression links with the original PPI's for the ICMg1 results in 14256 (OSR) and 15547 (DNAD) links in total. Missing values in the expression data were interpolated using the 10-nearest neighbor method [21].

#### Standard gene classes

For validation we derived standard gene class sets from the Gene Ontology [15] Biological Process annotations similarly as Shiga et al. [8]. We use the gene term annotation file of [16] (downloaded 29.2.2008), which extends the standard GO annotations to include all "part-of" and "is-a" annotations to give more comprehensive annotation data. GO annotations form a directed acyclic graph (DAG), with each node corresponding to a gene function (class label) to which the corresponding genes are assigned. Starting from the root of the DAG and proceeding from the parent to its children, we check the number of genes assigned to each node that appear in our data. The number of genes is reduced as we proceed in the DAG hierarchy. When we reach a node with size below a fixed cutoff value, we stop there and include its parent as a GO class in our standard class set. Nodes with more than 300 genes were omitted. We repeated this three times, using three different gene sets: 1) *All genes *in the data, 2) *Common genes *appearing in all Matisse runs, 3) *Total genes *appearing at least once in Matisse runs. Table 1 shows the number of standard classes *C *for both datasets (OSR: Osmotic shock response, DNAD: DNA damage) and the cutoff class size used for each three gene sets. Cutoff sizes were set to produce a bit more classes than Matisse found.

**Table 1:** Standard GO classes

Gene set	Cutoff size	*C *OSR	*C *DNAD
All genes	50	30	37

Common genes	40	27	29

Total genes	50	28	36

The obtained standard GO class sets, corresponding cutoff-sizes and the number of classes.

#### Protein complexes

We obtained a set of known protein complexes from the Comprehensive Yeast Genome Database at MIPS [22]. The total number of complexes in the used MIPS collection is 267. The number of protein complexes existing in our datasets with at least 2 proteins was 95 and 143 for OSMO and DNAD, respectively. Out of these, 33 and 46 contained at least five proteins.

### Validation measures

#### Perplexity

We measure the quality of the components by perplexity, which is a measure of the ability of a model to recover an underlying nominal category, and commonly used, e.g., in natural language processing. Perplexity is here applied to the confusion matrix formed of the evaluation samples, that is, to the table of frequencies with standard classes of the samples as columns (c), and the model-given components or clusters as rows (m). Perplexity for the evaluation sample is then defined as perp , where *N *is the number of evaluated data samples, indexed by *l*, and *c*_*l *_and *m*_*l *_are their class and component, respectively. The probabilities (*c*|*m*) are empirical probabilities, computed by normalizing the rows of the confusion matrix.

Perplexity is a monotonic function of the empirical conditional information *H*(*C*|*M*), and it can also be interpreted in terms of the average per-sample likelihood of a simple probability model formed from the table. For small sample sizes *N *both are upward-biased, because the  are computed from the same samples that we are evaluating. This is mostly a problem when one compares perplexities computed for different sizes of samples, whereas in our studies the sample sizes are the same. A remedy would be to use a leave-one-out version where sample *l *is not included in computing  for the evaluation of that particular sample.

Since we have only single assignments of nodes to clusters, we could use a bunch of other evaluation criteria for the clustering, such as the Normalized Mutual Information (NMI) used in [8]. But, as said, the likelihood in the perplexity corresponds to the conditional entropy, which in turn has been shown to be a good measure for clustering [23]. From the two-way measure proposed by Meilă, we only need the other "way", because the other corresponds to the fixed ground truth.

#### Gene Ontology enrichment analysis

In GO enrichment analysis a hypergeometric p-value is computed for each pair of found module and GO class [24]. Lower p-value means that the modules contain more of the same gene class than would be probable if they were generated randomly. A common approach is then to treat all pairs under a certain cutoff-value as enriched, and a higher number of enriched modules and GO classes is then considered as a better clustering. In our study we used the Fisher exact test and computed the number of enriched modules and GO classes on a range of p-values (*p *= {10^-1^,...,10^-10^}). The GO annotation data for yeast was downloaded 10.10.2008.

## Availability and requirements

Project name: ICMg;

Project home page: http://www.cis.hut.fi/projects/mi/software/ICMg/;

Operating system(s): Platform independent;

Programming languages: R, C;

License: GNU LGPL;

Any restrictions to use by non-academics: See GNU LGPL conditions.

## Authors' contributions

The authors developed the models and designed the experiments together. JP implemented the models and carried out the experiments. Both authors read and approved the final manuscript.
